# Identification of Clinically Relevant Yeasts from Avian Isolates Using API ID32C, MALDI-TOF MS, and ITS Sequencing: Potential Relevance from a One Health Perspective

**DOI:** 10.3390/vetsci13070615

**Published:** 2026-06-25

**Authors:** Begoña Acosta-Hernández, Nicolás Cabrera Guerle, Pablo Lorenzo García, Olga Armas Carballo, María del Mar Ojeda-Vargas, Victor Garcia-Bustos, Fernando Real Valcárcel, Soraya Déniz Suárez, Esther Licia Díaz Rodríguez, Inmaculada Rosario Medina

**Affiliations:** 1 Departamento de Patología Animal, Facultad de Veterinaria, Universidad de Las Palmas de Gran Canaria, 35413 Arucas, Spainfernando.real@ulpgc.es (F.R.V.); soraya.deniz@ulpgc.es (S.D.S.);; 2 Instituto Universitario de Sanidad Animal (IUSA), Facultad de Veterinaria, Universidad de Las Palmas de Gran Canaria, 35413 Arucas, Spain; victor.garcia123@alu.ulpgc.es; 3 Servicio de Microbiología, Complejo Hospitalario Universitario Insular Materno Infantil de Las Palmas de Gran Canaria, 35016 Las Palmas, Spain; mar.ojeda@ulpgc.es; 4 Departamento de Ciencias Clínicas, Facultad de Ciencias de la Salud, Universidad de Las Palmas de Gran Canaria, 35016 Las Palmas, Spain; 5 Severe Infection Research Group, Health Research Institute La Fe, 46026 Valencia, Spain

**Keywords:** Avian yeasts, MALDI-TOF, ITS sequencing, opportunistic yeasts, One Health, yeast identification

## Abstract

Wild and synanthropic birds harbour a diverse range of yeasts, including species of recognised clinical relevance. Given their close interaction with human activities, these birds represent a valuable source for investigating environmental yeasts and assessing the performance of identification methods. We investigated yeasts recovered from cloacal and crop samples of birds from Gran Canaria and compared routine identification methods with molecular sequencing. Twenty-four isolates were examined by biochemical profiling (API ID32C) and MALDI-TOF MS. Molecular identification based on ITS sequencing was carried out only for the sixteen isolates for which the two routine methods yielded discordant results, allowing resolution of their taxonomic identification. Phenotypic and proteomic methods showed limited agreement at the species level (8/24; 33.3%), with 16 discordant identifications resolved by sequencing. Pigeon isolates were dominated by members of the *Kazachstania telluris* complex, chiefly *K. bovina* (11/24; 45.8%), while partridges yielded taxa of clinical importance, including *Candida parapsilosis* and *Pichia kudriavzevii* (formerly *Candida krusei*). *Rhodotorula mucilaginosa*, *Debaryomyces* spp., and *Saccharomyces cerevisiae* were also detected. Comparative tests confirmed significant host-associated differences in species distribution (*p* < 0.05), and Cohen’s kappa indicated substantial agreement between API and MALDI-TOF at the genus level when benchmarked against ITS (κ = 0.71), although concordance was lower at the species level. In conclusion, these findings strengthen the case for integrating sequencing into diagnostic workflows, highlight the potential One Health relevance of yeast carriage by wild birds, and underscore the need for targeted surveillance at urban and game-handling interfaces where human exposure is likely.

## Simple Summary

Wild and urban-adapted birds can act as reservoirs and spreaders of yeasts that may affect human and animal health. In this study, we analysed cultivable yeasts recovered from pigeons and red-legged partridges in Gran Canaria and compared commonly used laboratory identification methods with DNA sequencing. Our results showed that traditional biochemical and proteomic techniques often failed to correctly identify some yeast species, whereas sequencing provided more reliable results. *Kazachstania bovina* was frequently recovered from pigeons, while clinically relevant species such as *Candida parapsilosis* and *Pichia kudriavzevii* were detected in partridges. Some of the identified species are recognised opportunistic pathogens and may be encountered through bird droppings or handling of game birds. Overall, this study supports the use of molecular methods for the identification of uncommon yeasts and highlights the value of including wildlife within a One Health surveillance initiative.

## 1. Introduction

Fungal diseases present an increasing challenge for medicine, veterinary health, and public health programmes, driven by growing numbers of susceptible individuals, antifungal resistance, and environmental change [1,2,3,4,5,6]. Birds may contribute to the maintenance and dispersal of environmental fungi, facilitating their distribution across urban and rural ecosystems through plumage, feet, and droppings [7,8,9,10,11,12,13].

Among bird species, urban pigeons (*Columba livia*) and red-legged partridges (*Alectoris rufa*) represent two distinct interfaces between wildlife, the environment, and human activities. Urban pigeons are abundant in cities, where their droppings create nitrogen-rich microhabitats that favour yeast growth and may harbour opportunistic pathogens such as *Cryptococcus*, *Candida*, *Rhodotorula*, and others [7,13,14,15,16,17,18]. Although red-legged partridges are not typically synanthropic, their handling during hunting activities may facilitate contact with environmental microorganisms by hunters and domestic animals.

The relevance of non-*Candida albicans* species, emergent *Kazachstania* within the *Kazachstania telluris* complex, and pigmented yeasts such as *Rhodotorula* has increased in recent years. This trend is supported by reports of invasive infections and reduced susceptibility to azoles and echinocandins [19,20,21,22,23,24,25,26,27,28,29,30,31,32]. At the same time, routine laboratory methods continue to struggle with uncommon or closely related taxa: API ID32C offers broad phenotypic coverage but often misassigns rare species [33,34], while MALDI-TOF accuracy depends on database breadth, yielding variable scores for environmental isolates [25,30,35,36,37,38].

Molecular identification of yeasts is commonly based on the analysis of ribosomal DNA (rDNA), which comprises conserved regions interspersed with more variable sequences. Among these, the internal transcribed spacer (ITS), including the ITS1 and ITS2 regions flanking the 5.8S rRNA gene, is widely accepted as the universal barcode for fungal identification due to its high interspecific variability combined with relative intraspecific conservation. This enables reliable discrimination of closely related taxa that are often indistinguishable by conventional approaches. In contrast, phenotypic identification systems rely on metabolic and morphological traits and may lead to misidentification, particularly among rare or closely related species. Similarly, although MALDI-TOF MS is a powerful tool for routine diagnostics, its accuracy depends on the completeness and quality of reference databases and may be limited for certain species complexes. Therefore, integrating molecular confirmation remains a practical strategy, particularly when surveillance targets unusual yeasts or when routine methods yield discordant results [19,30]. In this study, we analysed yeast isolates obtained from pigeons and partridges in Gran Canaria using API ID32C and MALDI-TOF, with ITS sequencing applied to isolates showing discordant identifications. Our aims were to describe the spectrum of species recovered, assess agreement between routine identification methods, and discuss their potential relevance from a One Health perspective. Molecular analysis was used to resolve discrepant results and to improve the characterisation of uncommon environmental yeasts recovered from avian hosts. This approach provided additional insight into the identification of uncommon avian yeasts in Gran Canaria.

## 2. Materials and Methods

### 2.1. Study Design and Setting

This study involved 85 birds (50 urban pigeons and 35 wild birds) obtained from two previous, unpublished undergraduate research projects conducted at our institution, from which 24 yeast strains were obtained. The isolates were obtained from 18 pigeons and 6 partridges. A cross-sectional design was applied using yeast isolates recovered from cloacal and crop samples of birds captured or collected across several municipalities in Gran Canaria (Spain). Urban pigeons were obtained from multiple municipalities: isolates 1–3 originated from Artenara; isolates 18, 19, and 24 from Agüimes; isolates 10–12 and 20–23 from Las Palmas de Gran Canaria; isolates 16–17 from Ingenio; and isolates 13–15 from Santa Lucía de Tirajana. Partridges were obtained from Agüimes (isolates 4–7) and Artenara (isolates 8–9). All samples were analysed at a single time point, with no repeated sampling of the same birds over time. Samples were collected using swabs with transport medium (Table 1).

Sampling of urban pigeons (*Columba livia*) was conducted in collaboration with municipal pest control programmes between November 2021 and May 2022. Wild red-legged partridges (*Alectoris rufa*) and wild pigeons (*Columba livia*) were obtained during the small game hunting season (September–October 2022). Samples were collected using swabs with transport medium (Eurotube^®^; Deltalab, Barcelona, Spain) and stored at 4 °C until processing at the Infectious Diseases Laboratory, Faculty of Veterinary Medicine, University of Las Palmas de Gran Canaria.

### 2.2. Microbiological Procedures

Swabs were streaked onto Sabouraud dextrose agar and incubated at 37 °C. Plates were examined after 24, 48, and 72 h of incubation, and colonies showing macroscopic characteristics compatible with yeasts were selected for further analysis. These colonies were subjected to Gram staining and microscopic examination, and those displaying yeast-like morphology were subcultured on fresh Sabouraud dextrose agar to obtain pure cultures. One or more yeast-like colonies could be recovered from each sample. When multiple yeast-like colonies were present, a single representative colony was selected for further analysis because the colonies showed similar macroscopic characteristics and comparable yeast-like morphology upon microscopic examination. Consequently, only one yeast isolate was ultimately obtained from each bird. In total, 24 yeasts were isolated and stored in brain–heart infusion (Merck KGaA, Darmstadt, Germany) supplemented with glycerol at −80 °C until further analysis.

### 2.3. Strain Processing

The strains were slowly thawed at room temperature. After homogenising the suspension, a loopful was removed and replated on Sabouraud dextrose agar. Plates were incubated at 30 °C, and to confirm the purity of the thawed strains, Gram staining and microscopic examination were performed.

### 2.4. Identification Methods

Phenotypic identifications were obtained using API ID32C galleries (bioMérieux, Madrid, Spain) following the manufacturer’s instructions and interpreted with APIWEB™ software version 1.4.1-3. Complementary API 20C AUX tests (bioMérieux) were performed on isolates previously identified as *Zygosaccharomyces* spp. by API ID32C in order to assess the reproducibility of the phenotypic identification.

Proteomic identification was carried out by the Microbiology Service of the Complejo Hospitalario Universitario Insular Materno-Infantil of Gran Canaria (CHUIMI) using a Bruker Microflex LT mass spectrometer with the MALDI Biotyper system (Bruker, Billerica, MA, USA). Yeast isolates were obtained from culture on solid agar media and prepared following the manufacturer’s instructions. An in-target extraction protocol [39] was applied, consisting of the addition of 70% formic acid directly onto the sample, followed by overlay with α-cyano-4-hydroxycinnamic acid matrix (Bruker Daltonics).

Spectra were acquired and analysed using the MALDI Biotyper software with the manufacturer’s reference database (version 4.1.100), which includes a wide range of clinically and environmentally relevant yeast species. The database contains reference spectra for multiple genera, including *Candida*, *Cryptococcus*, *Rhodotorula*, *Zygosaccharomyces*, and *Kazachstania*, among others. Identification results were generated based on score values according to the manufacturer’s criteria: ≥2.0, species-level identification; 1.7–2.0, genus-level identification; and <1.7, unreliable identification. Isolates with score values < 1.7 or discrepant results between identification methods were further analysed by sequencing.

Molecular confirmation was performed by Macrogen (Macrogen Inc.—Seoul, Republic of Korea). Sixteen isolates showing discordant identifications between API and MALDI-TOF were selected for sequencing. Genomic DNA was extracted using standard commercial protocols (performed by the sequencing provider). The internal transcribed spacer (ITS) region (ITS1–5.8S–ITS2) was amplified using universal primers ITS5 (5′-GGAAGTAAAAGTCGTAACAAGG-3′) and ITS4 (5′-TCCTCCGCTTATTGATATGC-3′). PCR products were sequenced using Sanger chemistry.

Consensus sequences were compared against the GenBank database using BLASTN (Versión: 2.13.0+), and taxonomic assignments were based on best-hit identity and query coverage, following current nomenclature. Sequence accession numbers are not available, as sequences were used exclusively for identification purposes.

### 2.5. Statistical Analysis

Statistical analyses were performed using the R software (version 4.3.0; R Foundation for Statistical Computing, Vienna, Austria). Frequencies and percentages were used to summarise species distributions by host and sample type. Differences in the proportions of selected taxa between pigeons and partridges were assessed using χ^2^ or Fisher’s exact test, as appropriate. Agreement between API and MALDI-TOF, each benchmarked against ITS (species-level match: yes/no), was quantified using Cohen’s kappa coefficient (κ) with 95% confidence intervals. Statistical significance was defined as *p* < 0.05. The analyses were intended to provide a simple inferential context for surveillance rather than exhaustive modelling [19,25,30].

## 3. Results

### 3.1. Isolates and Initial Identifications

Twenty-four yeast isolates were obtained from 85 birds. According to API ID32C, the most frequent preliminary assignments were *Zygosaccharomyces* spp. (11/24; 45.8%), *Cryptococcus humicola* (currently *Vanrija humicola*) (4/24; 16.7%), *Rhodotorula mucilaginosa* (4/24; 16.7%), *Saccharomyces cerevisiae* (1/24; 4.2%), *R. glutinis* (1/24; 4.2%), *Candida parapsilosis* (1/24; 4.2%), *Candida globosa* (1/24; 4.2%), and *Candida krusei* (currently *Pichia kudriavzevii*) (1/24; 4.2%). MALDI-TOF suggested *Kazachstania telluris*-complex identifications for most *Zygosaccharomyces*-labelled isolates and additionally recovered *Debaryomyces* spp., *Rhodotorula* spp., and *Naganishia*, among others (Table 1).

### 3.2. Sequencing Resolves Discordance

**Figure 1:**
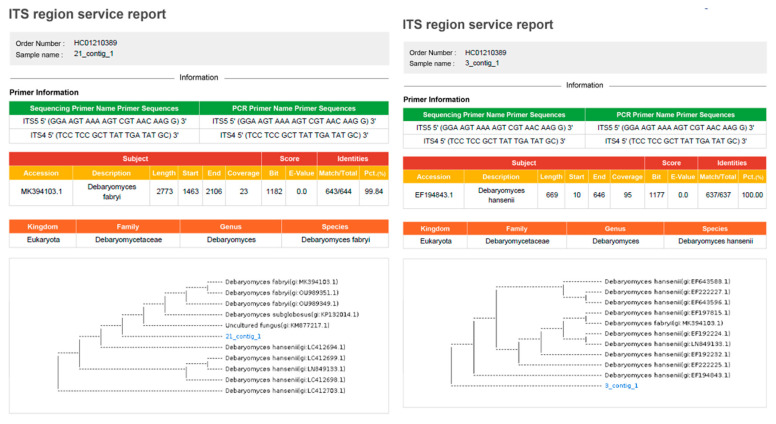
Representative ITS sequencing and BLASTN analyses showing robust identification of *Debaryomyces hansenii* and reduced query coverage for *D. hansenii* species complex (*D. fabryi*).

**Figure 2:**
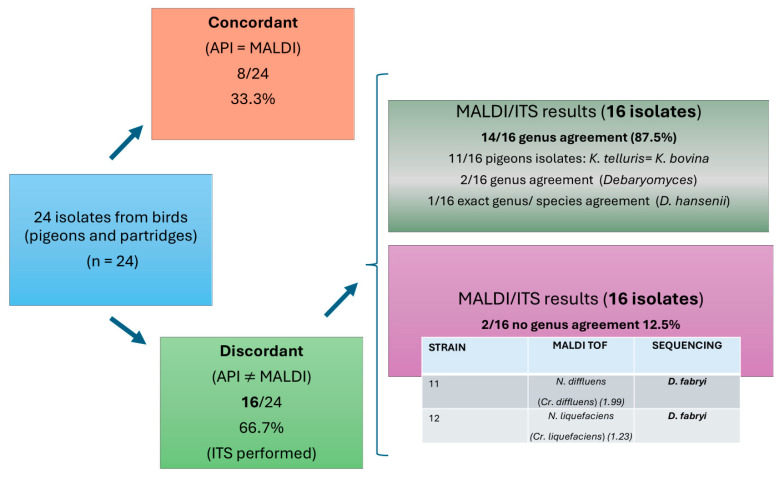
Flowchart of correct identifications and discordances by method (API, MALDI-TOF, and ITS).

ITS sequencing of 16 discordant isolates confirmed *K. bovina* in 11 cases (query coverage 99%) and *D. hansenii* in one case (query coverage 95%). Four additional isolates showed their best ITS match with *D. fabryi* (99.84% sequence identity), although the query coverage was limited (22–23%). Consequently, these isolates were assigned to the *D. hansenii* species complex, with *D. fabryi* representing the closest match. MALDI-TOF and API were fully concordant for seven isolates (*S. cerevisiae*, *R. mucilaginosa*, *Candida parapsilosis*, and *P. kudriavzevii*). Overall, exact species-level agreement between API and MALDI-TOF was 33.3% (8/24) (Table 1 and Table 2, Figure 1 and Figure 2).

### 3.3. Host-Associated Patterns

Pigeon isolates (*n* = 18) were dominated by *K. bovina* (11/18; 61.1%), whereas no *K. bovina* isolates were detected in partridges (*n* = 6). This difference was statistically significant (Fisher’s exact test, *p* = 0.007, χ^2^ = 6.76, *p* = 0.009). However, given the small sample size, these results should be interpreted with caution.

### 3.4. Concordance Analysis

Concordance between MALDI-TOF and ITS sequencing was evaluated in the subset of isolates showing discordant results between API ID32C and MALDI-TOF (*n* = 16). At the genus level, agreement was substantial (Cohen’s κ = 0.71), with correct identification in 14/16 isolates (87.5%).

However, at the species level, no agreement was observed, as only one of the identifications (sample 3) using MALDI-TOF matched the ITS results within this subset.

Due to the selective sequencing of discordant isolates, concordance could not be assessed for the full dataset (*n* = 24).

## 4. Discussion

This study aimed not only to characterise the diversity of yeasts carried by wild birds but also to evaluate the performance of different identification methods for clinically relevant and uncommon environmental yeasts. Our findings demonstrate that pigeons and partridges may harbour opportunistic yeasts of public health relevance, while also highlighting important discrepancies between phenotypic, proteomic, and molecular identification methods, particularly for uncommon taxa and members of the *Kazachstania telluris* complex.

In recent years, non-albicans and non-*Cryptococcus* yeast species have been identified in the cloaca and faeces of pigeons, including *Candida parapsilosis*, *S. cerevisiae*, and *Rhodotorula* spp., among others [40,41,42].

We were surprised to find that *P. kudriavzevii* accounted for 4.16% of our samples. Although this medically significant yeast has previously been reported in birds, including pigeon droppings [41], reports of *P. kudriavzevii* isolated from cloacal samples of red-legged partridges (*Alectoris rufa*) appear to be lacking in the available literature. The presence of this yeast in partridges may reflect environmental acquisition through feeding and contact with contaminated substrates, as *P. kudriavzevii* is widely distributed in natural and anthropogenic environments. As red-legged partridges are game birds that are frequently handled by hunters, their detection may be relevant from a One Health perspective in the context of environmental exposure. Identification results were concordant between MALDI-TOF and API ID32C, supporting the reliability of both methods for this species. *P. kudriavzevii* is an opportunistic pathogen associated with invasive candidiasis and has been reported to exhibit reduced susceptibility to fluconazole in previous studies [23,43,44,45,46,47]. It has also been reported in veterinary infections, including bovine mastitis [14,17,48,49,50].

API ID32C identified one isolate as *Candida globosa*, whereas MALDI-TOF and sequencing identified it as *D. hansenii* (formerly *Candida famata*), demonstrating concordance between proteomic and molecular approaches. Overall, *D. hansenii* represented 12% of the isolates identified by MALDI-TOF, all recovered from pigeon cloacas. Previous studies have reported variable accuracy of API ID32C for non-albicans *Candida* species, including misidentifications involving *Candida globosa* and related taxa [22,51,52,53]. Our findings reinforce the limitations of phenotypic systems for uncommon yeasts and support the use of MALDI-TOF or molecular approaches for more accurate identification. Although uncommon, *D. hansenii* has been associated with human infections and reduced susceptibility to fluconazole [9,10,29].

*Candida parapsilosis* accounted for 4.16% of isolates and was recovered from partridge droppings. Identification was concordant between API ID32C and MALDI-TOF. Although previously reported in pigeon droppings [41], this may represent the first description in partridge cloacal samples. *C. parapsilosis* is a recognised opportunistic pathogen associated with candidemia and healthcare-associated infections worldwide, with increasing antifungal resistance reported in several regions [6,11,24,31,54,55,56,57,58,59,60,61,62,63,64,65,66].

*Rhodotorula mucilaginosa* was the most frequent species isolated from partridges, whereas *R. glutinis* was detected in one pigeon sample. API ID32C and MALDI-TOF showed concordant identification for *R. mucilaginosa*, while *R. glutinis* was identified only at the genus level by MALDI-TOF. These yeasts have previously been associated with avian reservoirs [41,53] and are increasingly recognised as opportunistic pathogens in immunocompromised hosts [67,68,69]. Previous studies have also reported reliable MALDI-TOF performance for *Rhodotorula* spp., consistent with our findings [53]. In animals, they have been linked to infections in poultry, sheep, dogs, and cattle, highlighting their veterinary relevance [1,7,35,70,71].

*Saccharomyces cerevisiae* represented 4.16% of isolates, which were recovered from the pigeon crop and identified by both methods, consistent with previous reports in pigeon cloacas [41]. As this isolate was recovered from the crop rather than the cloaca, its presence may reflect recent ingestion or transient environmental exposure rather than gastrointestinal carriage. Although widely used in food production and as a probiotic, it rarely causes human infection [72,73]. We have not found any reports on animal health.

*Zygosaccharomyces* spp. accounted for 45.83% of isolates by API ID32C and have been previously reported in pigeon faeces [53], although earlier studies using API 20CAUX failed to detect them due to methodological limitations [74,75,76]. However, subsequent MALDI-TOF MS and ITS sequencing assigned these isolates to the *Kazachstania telluris* complex and specifically to *K. bovina*, highlighting the limitations of phenotypic identification methods for closely related yeasts. Human infections caused by members of the *K. telluris* complex are rare [2], whereas true *Zygosaccharomyces* species such as *Z. bailii* and *Z. rouxii* are mainly recognised for their resistance to preservatives and their role in food spoilage [37,77,78].

MALDI-TOF identified 9 of 11 *Zygosaccharomyces* isolates as belonging to the *K. telluris* complex. All 11 strains were identified as *Kazachstania bovina* by ITS sequencing and BLASTN analysis of PCR results. The assignment of these isolates to *K. bovina* was supported by ITS sequences showing 99% query coverage and 100% identity with reference sequences. Although the ITS data strongly supported species-level identification, additional molecular markers may provide greater taxonomic resolution within the *K. telluris* complex. These results support previous findings indicating that MALDI-TOF reliably identifies this complex but requires molecular methods for species-level resolution [4,51,79,80].

*Kazachstania* spp. are ubiquitous yeasts, with the *K. telluris* complex including *K. telluris*, *K. bovina*, *K. pintolopesii*, *K. sloofiae*, and *K. heterogenica*. Although human infections are rare, they can be invasive and may involve zoonotic transmission from pigeons [81]. Isolates initially identified as *Cryptococcus humicola* by API ID32C (16.66%) were reclassified as *D. hansenii* species complex and *K. bovina* using MALDI-TOF and PCR, highlighting the limitations of phenotypic methods and the need for molecular confirmation [51]. While *C. humicola* has been associated with human infections, including central nervous system involvement in HIV-positive patients [82,83,84,85,86], accurate identification is essential to properly assess its clinical relevance and antifungal resistance profile.

However, four isolates were assigned to the *D. hansenii* species complex based on ITS sequencing. Although *D. fabryi* represented the closest database match (99.84% sequence identity), the low query coverage obtained (22–23%) did not support a reliable species-level identification. Consequently, these isolates were conservatively classified within the *D. hansenii* species complex. This limitation is consistent with previous studies indicating that ITS-based identification may not provide sufficient resolution for discriminating closely related taxa within the *D. hansenii* complex and that additional loci or IGS-based analyses may be required for definitive species assignment [87,88]. In contrast, the single isolate identified as *D. hansenii* showed high ITS query coverage (95%), supporting a more robust taxonomic assignment. Additional discordance was observed with MALDI-TOF MS, which identified these isolates as *D. hansenii* (formerly *Candida famata*) or *Naganishia* spp., highlighting the limitations of routine identification methods and the influence of incomplete reference databases when analysing uncommon yeasts. The discordance observed between ITS sequencing and MALDI-TOF MS highlights the taxonomic complexity of the *D. hansenii* species complex. Members of this complex may have been historically underreported or misidentified owing to the limited discriminatory power of phenotypic and proteomic methods. *D. fabryi* has only relatively recently been recognised as a distinct species within the complex, which may have contributed to previous identifications as *D. hansenii* or *Candida famata* [34,87,88]. [34,87,88]. Although its clinical relevance remains unclear, it has been isolated from fermented and processed foods, suggesting a potential role in food contamination [89].

This study demonstrates that wild birds can harbour clinically relevant yeasts. Partridges carried *Candida parapsilosis* and *P. kudriavzevii*, whereas pigeons harboured isolates initially identified as *Cryptococcus humicola* by API ID32C, some of which were subsequently reclassified as *K. bovina* by PCR. However, these host-associated patterns should be interpreted with caution. Although all samples were collected and processed using a standardised protocol, the present study was not specifically designed to distinguish host-related effects from other factors such as geographical location, environmental conditions, or sampling context. Therefore, the observed distribution of yeasts cannot be attributed exclusively to host species. Within the *K. telluris* complex, MALDI-TOF MS identified isolates at the complex level but was unable to reliably discriminate species, requiring molecular confirmation for accurate identification [90]. This limitation likely reflects the close phylogenetic relationship and highly similar proteomic profiles among species such as *K. bovina*, *K. slooffiae*, and *K. pintolopesii*. The predominance of *K. bovina* in pigeons suggests that this species may be more widespread in avian reservoirs than previously recognised and may have been historically underreported due to phenotypic misidentification.

Overall, API ID32C showed acceptable performance for common clinically relevant yeasts such as *S. cerevisiae*, *Rhodotorula* spp., *Candida parapsilosis*, and *P. kudriavzevii*, whereas its performance was limited for uncommon environmental yeasts and closely related taxa, particularly within the *Kazachstania telluris* complex.

The biological significance of yeast recovery may also differ according to the sampling site. Yeasts isolated from crop samples may reflect recent ingestion or transient environmental exposure, whereas those recovered from cloacal samples may be more closely associated with gastrointestinal carriage and shedding. Consequently, the interpretation of yeast occurrence should take into account the anatomical origin of the sample when considering its ecological or epidemiological relevance.

**Limitations:** This study has several limitations that should be considered when interpreting the results. First, the sample size was relatively small (*n* = 24 isolates), which may limit the statistical power and generalisability of the findings. In addition, the number of samples was unevenly distributed between host species, which may have affected comparisons between pigeons and partridges.

A second limitation of this study is that MALDI-TOF MS identification was performed using an on-target formic acid extraction protocol rather than a full protein extraction procedure. Although this approach is widely used in routine diagnostics, it may reduce identification accuracy for uncommon or closely related yeasts, particularly when commercial reference databases are incomplete.

A third limitation is that molecular sequencing was selectively performed only for isolates showing discrepant results between identification methods. Consequently, not all isolates were confirmed by ITS sequencing, which may have introduced selection bias and limited the ability to assess the overall concordance and diagnostic accuracy of the evaluated methods across the full dataset.

## 5. Conclusions

This study demonstrates that wild pigeons and red-legged partridges in Gran Canaria harbour diverse yeast communities. *K. bovina* predominated in pigeons, suggesting a role in its environmental dissemination, whereas partridges carried clinically relevant species such as *Candida parapsilosis* and *P. kudriavzevii*. To our knowledge, reports of these yeasts in cloacal samples of red-legged partridges are scarce.

The findings also highlight the limitations of phenotypic identification methods when applied to uncommon environmental yeasts and support the value of molecular approaches for resolving discrepant results and improving taxonomic accuracy.

The relatively small number of isolates analysed and the selective application of ITS sequencing should be considered when interpreting these findings. Nevertheless, the study provides additional information on the diversity of avian-associated yeasts in Gran Canaria and contributes to current knowledge of uncommon yeasts recovered from wild birds. Further investigations involving larger sample sizes, broader geographic coverage, and additional molecular markers would help to better characterise the ecology and diversity of these yeasts and to clarify their significance at the wildlife–environment interface.

## Figures and Tables

**Table 1:** Agreement of API ID32C and MALDI-TOF against ITS sequencing (selected isolates).

Strain	Bird Sample Type)	API ID32C Identification/% ID	API Profile	MALDI-TOF MS Identification	Score	Sequencing Result
1	Wild pigeon (crop)	*S. cerevisiae*/99.8%	4 0 6 0 0 0 0 0 0 1	*S. cerevisiae*	1.86	No
2	Wild pigeon (cloaca)	*Cr. humicola* *(V. humicola)*/91.8%	5 7 7 7 7 7 7 7 1 7	*D. hansenii (C. famata)*	1.31	*D. hansenii* species complex
3	Wild pigeon (cloaca)	*C. globosa*/99.8%	4 1 0 6 0 0 0 0 1 1	*D. hansenii (C. famata)*	1.56	*D. hansenii*
4	Partridge (cloaca)	*R. mucilaginosa*/65%	4 0 2 1 7 0 0 0 0 1	*R. mucilaginosa*	2.22	No
5	Partridge (cloaca)	*R. mucilaginosa*/65%	4 0 2 1 7 0 0 0 0 1	*R. mucilaginosa*	2.20	No
6	Partridge (cloaca)	*C. parapsilosis*/80.4%	5 5 4 6 3 4 0 3 1 7	*C. parapsilosis*	2.00	No
7	Partridge (cloaca)	*C. krusei* *(P. kudriavzevii)*/98.3%	0 3 0 0 0 0 0 0 0 3	*P. kudriavzevii*	2.04	No
8	Partridge (crop)	*R. mucilaginosa*/65%	4 0 2 1 7 0 0 0 0 1	*R. mucilaginosa*	2.32	No
9	Partridge (crop)	*R. mucilaginosa*/65%	4 0 2 1 7 0 0 0 0 1	*R. mucilaginosa*	2.28	No
10	Urban pigeon (cloaca)	*Zygosaccharomyces* spp./56.5%	0 0 0 0 0 0 0 0 0 1	*K. telluris*	1.93	*K. bovina*
11	Urban pigeon (cloaca)	*Zygosaccharomyces* spp. /56.5%	0 0 0 0 0 0 0 0 0 1	*N. diffluens* *(Cr. diffluens)*	1.99	*D. hansenii* species complex
12	Urban pigeon (cloaca)	*Cr. humicola* *(V. humicola)*	5 7 7 7 7 7 7 7 1 7	*N. liquefaciens* *(Cr. liquefaciens)*	1.23	*D. hansenii* species complex
13	Urban pigeon (cloaca)	*Zygosaccharomyces* spp. /56.5%	0 0 0 0 0 0 0 0 0 1	*K. telluris* complex	1.76	*K. bovina*
14	Urban pigeon (cloaca)	*Zygosaccharomyces* spp. /56.5%	0 0 0 0 0 0 0 0 0 1	*K. telluris* complex	2.09	*K. bovina*
15	Urban pigeon (cloaca)	*Zygosaccharomyces* spp. /56.5%	0 0 0 0 0 0 0 0 0 1	*K. telluris* complex	2.01	*K. bovina*
16	Urban pigeon (cloaca)	*Zygosaccharomyces* spp. /56.5%	0 0 0 0 0 0 0 0 0 1	*K. telluris* complex	2.07	*K. bovina*
17	Urban pigeon (cloaca)	*Cr. humicola* *(V. humicola*)/91.8%	5 7 7 7 7 7 7 7 1 7	*K. telluris* complex	2.03	*K. bovina*
18	Urban pigeon (cloaca)	*Zygosaccharomyces* spp. /56.5%	0 0 0 0 0 0 0 0 0 1	*K. telluris* complex	1.98	*K. bovina*
19	Urban pigeon (cloaca)	*Cr. humicola* *(V. humicola)*/91.8%	5 7 7 7 7 7 7 7 1 7	*K. telluris* complex	1.88	*K. bovina*
20	Urban pigeon (cloaca)	*R. glutinis*/98.2%	4 0 6 3 5 5 0 3 0 1	*Rhodotorula* spp.	1.27	No
21	Urban pigeon (cloaca)	*Zygosaccharomyces* spp. /56.5%	0 0 0 0 1 0 0 0 0 0	*D. hansenii (C. famata)*	1.64	*D. hansenii* species complex *i*
22	Urban pigeon (cloaca)	*Zygosaccharomyces* spp. /56.5%	0 0 0 0 0 0 0 0 0 1	*K. telluris* complex	1.86	*K. bovina*
23	Urban pigeon (cloaca)	*Zygosaccharomyces* spp. /56.5%	0 0 0 4 0 0 0 0 0 1	*K. telluris* complex	2.07	*K. bovina*
24	Urban pigeon (cloaca)	*Zygosaccharomyces* spp. /56.5%	0 0 0 0 0 0 0 0 0 1	*K. telluris* complex	1.92	*K. bovina*

NOTE: API profiles correspond to numerical codes generated by the API ID32C system. MALDI-TOF MS scores were interpreted according to the manufacturer’s criteria: ≥2.0 indicates species-level identification; 1.7–2.0 indicates genus-level identification; and <1.7 indicates unreliable identification. Sequencing of the ITS region was used as the reference method when available. “No” indicates that sequencing was not performed. Abbreviated genera: *S*., *Saccharomyces*; *Cr*., *Cryptococcus*; *C*., *Candida*; *V*., *Vanrija*; *R*., *Rhodotorula*; *P*., *Pichia*; *D*., *Debaryomyces*; *K*., *Kazachstania*; *N*., *Naganishia*.

**Table 2:** ITS sequencing results and BLASTN analysis of yeast isolates showing discordant identification between API ID32C and MALDI-TOF MS.

Isolate	MALDI-TOF ID	ITS Identification	% Identity	Query Coverage (%)	Accession
2	*Debaryomyces* spp.	*D. hansenii* species complex	99.8	23	MK394103.1
3	*D. hansenii*	*D. hansenii*	100.0	95	EF194843.1
10	*K. telluris* complex	*K. bovina*	100.0	99	KY103626.1
11	*Debaryomyces* spp.	*D. hansenii* species complex	100.0	22	MK394103.1
12	*Debaryomyces* spp.	*D. hansenii* species complex	99.8	22	MK394103.1
13	*K. telluris* complex	*K. bovina*	100.0	99	KY103626.1
14	*K. telluris* complex	*K. bovina*	100.0	99	KY103626.1
15	*K. telluris* complex	*K. bovina*	100.0	99	KY103626.1
16	*K. telluris* complex	*K. bovina*	100.0	99	KY103626.1
17	*K. telluris* complex	*K. bovina*	100.0	99	KY103626.1
18	*K. telluris* complex	*K. bovina*	100.0	99	KY103626.1
19	*K. telluris* complex	*K. bovina*	100.0	99	KY103626.1
21	*Debaryomyces* spp.	*D. hansenii* species complex	99.8	23	MK394103.1
23	*K. telluris* complex	*K. bovina*	100.0	99	KY103626.1
24	*K. telluris* complex	*K. bovina*	100.0	99	KY103626.1

NOTE: ITS sequencing was performed for isolates showing discordant results between API ID32C and MALDI-TOF MS. Identification was based on BLASTN analysis against the GenBank database. The closest match is reported with percentage identity, query coverage, and accession number. MALDI-TOF identifications are shown for comparison. Species names follow current taxonomy.

## Data Availability

The original contributions presented in this study are included in the article. Further inquiries can be directed to the corresponding authors.
